# Dr. Currie's Medical Reports

**Published:** 1803-01-01

**Authors:** J. Currie

**Affiliations:** Liverpool


To Dr. BATTY.

Sir,

WILL you have the goodness to signify, in your next Journal, that I am preparing a third edition of my Medical Reports for the press, in which there will be considerable additions; and that I solicit the Communications of such gentlemen as may have made a trial of the practice in fever, which I have recommended. Mr. Nagle, late late Surgeon of his Majesty's ship Ganges, in a letter dated Torbay, 22d of last September, informed me of the extraordinary success with which he had used the cold affusion in what he calls the endemial causus of the West Indies, from which quarter he was just arrived. With the view of obtaining more particular information, I addressed a few queries to him at Portsmouth; but not having received an answer, I conclude that my letter has not reached him, through the insufficiency of the address.—Wishing much to hear again from that gentleman, and not knowing where he resides, I take the liberty of signifying this wish to him through the medium of your Journal. I am, &c.

*Liverpool*, 12 *Dec.* 1802. J. CURRIE.

P.S. It would give me great pleasure to hear from any of the respectable practitioners of our fleets or armies, who may have made trial of water internally or externally in fevers, or febrile diseases, in any quarter of the world.

